# Vitamin A Supplementation Coverage and Ocular Signs among Children Aged 6–59 Months in Aleta Chuko Woreda, Sidama Zone, Southern Ethiopia

**DOI:** 10.1155/2021/8878703

**Published:** 2021-04-23

**Authors:** Temesgen Nigusse, Achamyelesh Gebretsadik

**Affiliations:** ^1^Aleta Chuko Woreda Health Office, Hawassa, Sidama, Ethiopia; ^2^School of Public Health, College of Medicine and Health Sciences, Hawassa University, Hawassa, Ethiopia

## Abstract

**Background:**

Periodic vitamin A supplementation to children is a cost-effective strategy to avert vitamin A deficiency. However, few pieces of evidence are available about the coverage of vitamin A supplementation at the community level in the study area. Therefore, the aim of this study was to assess vitamin A supplementation coverage and prevalence of ocular signs of vitamin A deficiency among children aged 6–59 months.

**Methods:**

Community-based cross-sectional study design was conducted using a two-stage stratified random sampling method. Data were collected from mothers with children aged 6–59 months using a structured pretested questionnaire. A total of 665 children aged 6 to 59 months were examined for clinical signs and symptoms of vitamin A deficiency by trained clinical health professionals. Descriptive statistics and logistic regression were done.

**Result:**

Vitamin A supplementation coverage in the study area was 36.2% (95% CI: 32.6–39.9). Overall, the prevalence of xerophthalmia was 2.7%. Age group 6–23 months (AOR: 2.1, 95% CI: 1.4–2.9), good maternal knowledge (AOR: 1.5, 95% CI: 1.2–2.1), children with high wealth status (AOR: 2.3, 95% CI: 1.4–3.8), precampaign health education on vitamin A (AOR: 3.4,95% CI: 2.1–5.6), member of Health Development Army (AOR: 2.7, 95% CI: 1.7–4.2), and access to health facility within <30 minutes (AOR: 2.5, 95% CI: 1.6–3.8) were significantly associated with the receipt of vitamin A capsule.

**Conclusion:**

Vitamin A supplementation coverage of the study area was low as compared to the UNICEF threshold of 70%. Vitamin A deficiency is a public health problem in the study area. Increasing maternal level of knowledge, precampaign health education on vitamin A supplementation, and strengthening Health Development Army are recommended to increase the vitamin A supplementation coverage.

## 1. Background

Vitamin A (retinol) is a fat-soluble micronutrient needed in small amounts by humans and plays diverse roles in human physiology, ranging from vision pigment formation to regulating the expression of genes for growth hormones [1]. An insufficient amount of vitamin A uptake decreases children's ability to fight childhood infection and increases the risk of childhood death and visual impairments [2–4].

Biannual vitamin A supplementation (VAS) program was advocated by the World Health Organization (WHO) since the 1990s in the high vitamin A-deficiency (VAD) settings [5]. In 1995, the Ethiopian Ministry of Health and United Nations International Children's Emergency Fund (UNICEF) began implementing universal VAS through oral delivery of vitamin A using the Expanded Program on Immunization (EPI) [2]. In 2004, Ethiopia adopted the program as a main child survival strategy and started to supplement vitamin A to targeted children through enhanced outreach services twice a year [6]. Due to the low access to vitamin A-rich food, preschool children and pregnant mothers are the most at-risk groups for vitamin A deficiency (VAD) mainly in Africa and in Southeast Asia [4, 5].

In Africa, 56.4 million (44.4%) and 2.55 million (2%) of preschool children are affected by subclinical VAD and night blindness, respectively. And more than one-third of the global xerophthalmia burden was reported in Africa [7].

In sub-Saharan Africa, 73% of children received at least one dose of vitamin A. However, millions of children are still not fully protected with the recommended biannual doses [8]. VAS and treatment in children between 6 and 59 months of age in the developing countries reduce the risk of all-cause mortality by 25% and diarrhea-associated death by 30% [8].

National VAS coverage reached 95% in 2008/2009 year [9]. However, the national VAD survey report indicated that the prevalence of subclinical VAD and Bitot's spot of 37.7% and 1.7%, respectively [10], which was much higher than the cut-off point set by WHO, which is 1% for night blindness and 0.5% for Bitot's spot. According to the 2016 Ethiopian micronutrient survey report, the national vitamin deficiency estimated based on retinol adjusted for inflammation among children aged 6–59 months was found to be 13.9% [11].

The benefits of VAS in child's survival have been well demonstrated in a different part of the world [12]. In the southern part of Ethiopia, few studies were done to measure vitamin A supplementation coverage at the community level. However, most of them are done more than ten years ago [13, 14] and do not address the ocular sign prevalence. To the best of the authors' knowledge, only one recent study was carried out in the other corner of southern Ethiopia. The study revealed that two-thirds of children between 6 and 59 months of age have received VAS [15]. Therefore, this study aimed to assess the current VAS coverage and ocular signs among children aged 6–59 months in the study area.

## 2. Methods

### 2.1. Study Area

Woreda is the third-level administrative division of Ethiopia and subdivided into several kebeles, which is the smallest unit of local government in Ethiopia. Aleta Chuko woreda is one of the 36 woredas in the Sidama administrative zone of Southern Nations, Nationalities, and Peoples' Regional State (SNNPRS) of Ethiopia. The zone is bordered on the north and east by Oromia region, on the south by the Oromia region and Gedeo Zone, and on the west by Bilate river, which separates it from Wolaita Zone. The projected population in 2019 was 203,142 being children of 6–59 months (28,317). The woreda has 24 rural and 5 urban kebeles. The main staple foods of the area are inset “false banana,” maize, fruits, and vegetables. Concerning the health care services, there are one woreda hospital, 7 health centers, 24 health posts, and 7 private clinics. VAS is conducted in the woreda through the Community Health Day (CHD) program by Health Extension Workers every six months. In CHDs, the Health Extension Workers (HEWs) mobilize the community with children under 5 years to come to the health post for malnutrition screening, deworming, and vitamin A capsule supplementation [16]. The last VAS was conducted in the area two months preceding this survey.

### 2.2. Study Design and Period

A community-based cross-sectional study design was conducted between 15 December 2018 and 15 January 2019.

### 2.3. Study Population

Mothers with children aged 6–59 months that live in the area for at least six months prior to the survey were included in the study.

### 2.4. Exclusion Criteria

Mothers with children aged 6–59 months who were sick, had a mental problem, and live in the area for less than six months were excluded.

### 2.5. Sample Size Estimation

The sample size was calculated for vitamin supplementation coverage, associated factors, and prevalence of conjunctival xerosis with a child receiving vitamin A capsule using both single- and double-population formula. Finally, comparing the three objectives' sample size, the largest sample size 673 was taken, which was determined by using a single-proportion formula *n* = *Z*^2^ (1 − *α*) *p* (1 − *p*)/(se)^2^. Desired confidence interval = 95% (*Z* = 1.96), level of precision (marginal error) = 3%, non–response rate and missing data = 10%, design–effect = 1.5, and *P*=89.3% of vitamin A supplementation coverage were taken from the Southern Nations, Nationalities, and Peoples' Regional State health bureau report of 2017 [17].

### 2.6. Sampling Procedure

A two-stage stratified sampling procedure was employed for sample selection. The woreda has been stratified by their administrative kebeles, and accordingly 4 rural and 2 urban kebeles were selected for the study by using lottery methods. The total numbers of children of 6–59 months living in these selected kebeles were obtained from the local administration. The proportional allocations to the size of each selected kebeles were conducted. After a proportional allocation to each kebele, *k*th or interval was calculated for each kebele and systematic sampling technique was employed to reach the study subjects. From each household, one study subject was selected, and where there is more than one eligible study subject in the household only one child was included by a lottery method. In a situation where eligible study subject temporarily absent at the selected house, two repeated visits were done; otherwise, an adjacent house was substituted.

### 2.7. Data Collection Method

Data were collected from mothers with children aged 6–59 months using a structured questionnaire. A total of 8 trained senior clinical health professionals and two supervisors who speak the local language fluently participated in data collection. At the time of the interview, the vitamin A capsule was demonstrated to the interviewer to facilitate recall of whether the child received or did not receive preceding the survey. The two-day training was given for data collectors by one principal investigator and one optometrist with field practice. Five percent of the questionnaire was pretested in the other kebeles that were not selected for the study. The pretest result was not included in the final analysis. Site selection for clinical assessments and a few frightened children were observed as a challenge during a pretest. To minimize these problems during the actual data collection, clinical assessments were done under an available shade with natural lightening and the examination was carried out after the frightened child calmed down. Eyes of eligible (665) children were examined by trained clinical health professionals for ocular signs of VAD such as Bitot's spot, conjunctival xerosis, corneal ulcers, corneal scar, and corneal xerosis, by using an ophthalmoscope, a pen torch, and magnifiers (fixed hand lens). History of the night blindness of children was obtained by asking mothers. To ensure the validity and reliability of the procedure, all children with ocular sign and symptoms were reexamined by a senior optometrist. And then, children with ocular sign of VAD were taken only for those children who were examined and confirmed by the senior optometrist. The pretest data were also entered to SPSS, and reliability test was done for nine items that were used for measuring maternal knowledge. The result showed a Cronbach's *α* of 0.77.

### 2.8. Data Management and Analysis

Data were entered and analyzed using SPSS version 20. Data cleaning was performed to check for inconsistencies and missing values and variables. Data were checked daily for incompleteness and inconsistency by two field supervisors and one principal investigator. From the total houses included for an interview, five percent of them were randomly visited by field supervisors to check data quality. Descriptive analysis like mean and standard deviation were determined for continuous variables after checking the distribution of the data for its normality. The household wealth index was calculated using household properties. Principal component analysis (PCA) was used to compute the household wealth index and categorized into the lowest, middle, and highest. The overall knowledge level of mothers on VIT A was assessed using nine questions and each had one point with a total of nine marks. Each score for each question was calculated and summed up to give the overall knowledge. Pearson's chi-square test was also done to measure the association between vitamin A deficiency and ocular sign.

Logistic regression was used to see the independent effect of predictors on outcome variables (VIT A utilization). Independent predictors that had a *P* value less than 0.25 at binary logistic regression were transferred to multivariable analysis. Crude odds ratio (COR) and the adjusted odds ratio (AOR) were presented at a confidence interval of 95%.

Multicollinearity effect was checked by using a linear diagnostic test of mean-variance inflation factor (VIF) at cut-off point >10. The model goodness-of-fit was tested by using the Hosmer–Lemeshow test at *P* value>0.05 as the best fit. All tests were two-sided and variables with *P* < 0.05 in the final multivariable model were considered as independent predictors of vitamin A utilization.

### 2.9. Operational Definition

#### 2.9.1. Vitamin A-Supplemented Children

A child who was given vitamin A capsule in the preceding 6 months of the survey as reported by the mother (after the mother is shown the capsule) [18].

#### 2.9.2. Vitamin A-Deficient Child

A child who was examined by a trained clinical health professional for ocular signs of VAD (Bitot's spot, conjunctival xerosis, corneal ulcer, corneal scar, and corneal xerosis) during data collection and unable to see in dim light or dark and has ocular symptoms of vitamin A deficiency as well.

### 2.10. Knowledge Measurement

#### 2.10.1. Good Knowledge on VIT A

A respondent who correctly answered 6 (>60%) and above questions among nine knowledge-related questionnaires on VIT A [19].

#### 2.10.2. Poor Knowledge on VIT A

A respondent who correctly answered ≤5 (<60%) questions among nine knowledge-related questions on VIT A [19].

#### 2.10.3. Health Development Army (HDA)

It is a one to five networks of women volunteers organized to promote health and prevent disease through community participation and empowerment. It also helps to identify local salient bottlenecks that hinder families from utilizing maternally and child service [20].

#### 2.10.4. Model Household

It is a household measured in 18 health extension packages by Health Extension Workers (HEW) and verified as a model or non–model household.

## 3. Results

### 3.1. Sociodemographic Characteristics

A total of 665 mothers–children pairs were included in the study with a response rate of 98.8%. Of the total respondents, 625 (94%) were biological mothers and the rest 40 (6%) were caregivers. The mean age of the mother was 26.5 (±4.62 SD). Among the total respondents, 619 (93.1%) had attained formal education. Four hundred sixty-seven (70.2%) of respondents said that they were living in less than 30-minute walking distance from the nearest health institution (Table 1).

### 3.2. Demographic Characteristics of Study Children

Four hundred twenty-five (63.9%) of the children were between the ages of 24 and 59 months and the mean age (±SD) was 29.31 (±13.8) and 334 (50.2%) were female children (Table 2).

### 3.3. Vitamin A-Related Awareness of Mothers

**Figure 1:**
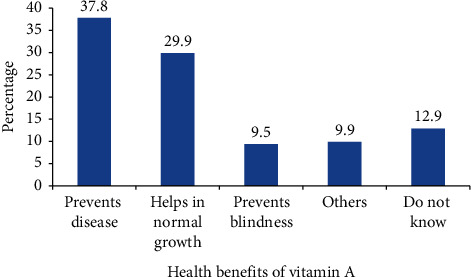
Health benefits of VIT A in Aleta Chuko woreda, 2019.

Out of 665 respondents, 155 (23.3%) heard about VAS. The main sources of information were from health post 62 (40.1%) and health center 57 (36.7%). The rest got VAS information from immunization session 17 (10.9%), Health Development Army 8 (5.2%), and radio or television 11 (7.1%). Of all, 561 (84.4%) did not get health education on VAS in the past six months. Among 513 (77.1%) respondents that heard about vitamin A, 194 (37.8%) are aware of the fact that vitamin A prevents disease while 66 (12.9%) did not mention any of its health benefits (Figure 1).

Four hundred seventy-one (71.2%) of the respondents listed one or more natural food sources of vitamin A among the total participants. The number of respondents that heard about vitamin A deficiency was 334 (50.2%). Almost half, 326 (49%) of the respondents listed one or more consequences of VAD. Three hundred eighty-two (57.4%) of the respondents correctly identified vitamin A capsule has a blue color among different similar drugs. Out of all, 150 (22.6%) showed good knowledge of VIT A (Table 3).

### 3.4. Health Seeking Behavior

Six hundred twenty (93.2%) of the respondents had at least one antenatal care for the child selected for the study and 529 (79.5%) said that they gave birth in health facilities. Postpartum supplementation of VAS in the study area is 166 (25%). Above half, 360 (54.1%) of the study participants had participated in the pregnant mother forum. One hundred sixty-seven (25.1%) and 108 (16.2%) of the study participants were a member of the Health Development Army and model in health extension package, respectively (Table 4). More than half 364 (54.7%) of the mothers prefer to get VAS home to home and 242 (36.4%) at health institution.

### 3.5. Vitamin A Supplementation Coverage

Of the total children aged 6–59 months, 241 (36.2%) with 95% CI (32.6–39.9) received vitamin A capsules in the preceding six months of the survey. Among VIT A-supplemented children, 122 (50.6%) of the children got vitamin A supplementation through Community Health Day (CHD), whereas 96 (39.8%) got it at immunization sessions (Table 5).

There was variation among the age group in taking vitamin A capsules. One hundred eight (44.8%) of the children that took vitamin A capsules in the preceding six months of the survey were found in age groups 6–23 months and 133 (55.2%) at the age of 24–59 months (Table 6).

### 3.6. The Reason Why a Child Did Not Take VIT A

**Figure 2:**
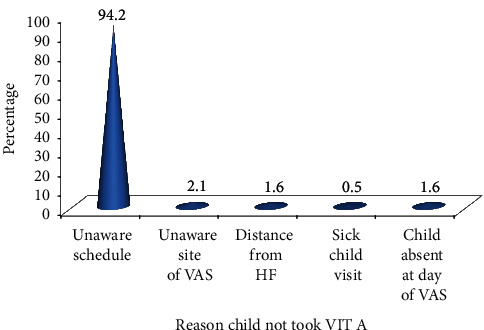
Reasons mentioned by mothers why the child did not take VIT A supplement in the preceding six months of the survey in Aleta Chuko woreda, SNNPR, 2019.

Of the total study participants, 424 (63.8%) children did not receive VAS in the preceding six months of the survey. The main reason described by mothers why their child did not take VAS was “unaware of schedule” 399 (94.2%) (Figure 2).

### 3.7. Prevalence of Ocular Signs of VIT A Deficiency

**Figure 3:**
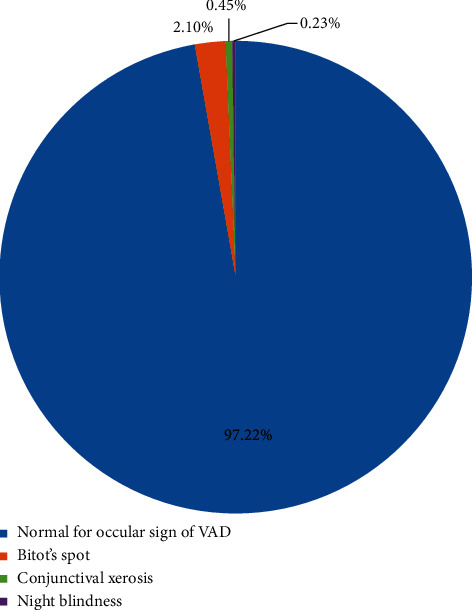
Prevalence of ocular signs of vitamin A deficiency among children aged 6–59 months in Aleta Chuko woreda, SNNPR, 2019.

Among all (665) children examined for ocular signs of vitamin A deficiency, the prevalence of Bitot's spot account for 14 (2.1%) and conjunctival xerosis 3 (0.45%). Of the total (425) 24–59-month children assessed for history of night blindness by asking their mothers, the prevalence was 1 (0.23%). The overall prevalence of xerophthalmia was 18 (2.7%) (Figure 3). Chi-square test result also showed significant association between VAT A supplement and ocular sign (2 = 7.41, *P* value = 0.006).

### 3.8. Factors Associated with the Utilization of VAS

The odds of receiving vitamin A capsules were two times more likely among children aged 6–23 months than 24–59 months of children (AOR: 2.1, 95% CI: 1.4–2.9). Children whose mothers had good knowledge of vitamin A have received vitamin A capsule 1.5 times more likely than children from poor-knowledge mothers (AOR: 1.5, 95% CI: 1.2–2.1). The children from high-wealth-status families have received vitamin A capsule 2 times more likely compared to children from poor families (AOR: 2.3, 95% CI: 1.4–3.8) and middle family who received vitamin A capsule nearly three times more likely than children from poor family (AOR: 2.7, 95% CI: 1.6–4.5). Study participants who got health education on VAS in the previous last six months before the study utilized VAS three times more likely than those of the family who did not get health education in the last six months before the study (AOR: 3.4, 95% CI: 2.1–5.6) (Table 7).

## 4. Discussion

This study aimed to assess the coverage of VAS and the prevalence of ocular signs in children aged 6–59 months. Accordingly, this study found that 36.2% VAS coverage and 2.1% prevalence of Bitot's spot of VAD. Age of children, high wealth index of the family, preference of VAS methods, level of maternal knowledge, and precampaign health education on VAS are statistically significant factors associated with utilization of VAS.

Our study revealed that vitamin A supplementation coverage in the study area was low as compared to the recommended coverage of 70% by UNICEF and Ethiopian National Micronutrient Survey report of 2016, which was 57.9% [8, 21]. There was also low coverage than that of the finding from Boloso Sore woreda of Wolaita Zone (83.1%) [14]. This might be due to the fact that the majority (84.4%) of this study participants did not get health education on VAS before the campaign, lack of awareness on the recommended schedule of VAS, unaware of who were eligible children for VAS, unaware frequency of VAS, and most of the mothers preferred to get VAS through home-to-home visit.

The prevalence of Bitot's spot in the study area was slightly higher than that of the findings (1.46%) from north Ethiopia [22], national prevalence (1.7%), SNNPR [10], and the WHO cut-off point of 1.56% [3]. This might be as a result of very low coverage of VAS and variation in the study period. However, our finding was lower than that of the prevalence (2.9%) reported from Demba, northwest Ethiopia [23]. This might be due to sampling size, timing, and cultural difference.

Many children with VAD do not develop ocular signs of VAD (xerophthalmia) within a short period of time, thus detecting ocular signs of VAD called as “tip of the iceberg” [24]. However, our finding showed that the ocular signs of VAD are a public health problem in the study area.

There was a significant variation among children of different age groups in taking vitamin A supplementation. In this study, children aged 6–23 months were more supplemented with vitamin A than children aged 24–59 months. This could be due to the fact that this age group got the first dose of vitamin A capsule with pentavalent-3 at six months and measles vaccine at nine months. This finding is in line with the studies done in Ghana and India [25]. This might be because in India routine immunization was administered up to 24 months with VIT A and the Ghana study revealed that it is as a result of poor commitment of health workers in informing parents about VAS [26]. On the other hand, the EDHS of 2011 report shows no variation among different age groups in taking VAS [27].

In this study, children from middle- and high-wealth-status households had a higher likelihood to receive VAC supplement as compared to their counters. This finding is similar to a study done in Nigeria [28]. This might be because those people who are of the lowest economic level might have limited access to the service because of lack of modern information and transportation.

In this study, children of mothers who had good knowledge of vitamin A supplementation to their children were more likely to receive VAS supplement compared with their counterparts. This finding was similar to a study conducted in Ghana [26].

Health Development Army is an unpaid 1-to-5 female volunteer's network team launched by EFMoH in 2011 to empower women with 18 health extension packages through Health Extension Workers' guidance and supervision. This team discuss and exchange health information with their families and community and actively participate in health service utilization [29–31].

In this study, being a member of HDA has been significantly associated with receipt of VAS. This finding was supported by a study conducted in the Yeky district of southwest Ethiopia [32] and in four regions of Ethiopia [33]. In our findings, those study participants who were members of HDA might have developed a good awareness to utilize maternal and child health services.

Taking health education regarding VAS in different sources in the last six months preceding the supplementation was significantly associated with VAS coverage. Our finding had similarities with a pooled study conducted on the determinants of good VAS coverage in thirteen sub-Saharan Africa countries by taking data of 2010–2015 [34], Benghazi (Libya) [35], and Kenya [36].

Children whose mothers had access to reach nearby health institutions within 30 minutes were significantly associated with VAS coverage. This was consistent with the postevent coverage survey report of VAS and deworming in Tanzania [37]. Our finding was inconsistent with the study done in Ghana [26]. This is because in Ghana, static and outreach child welfare clinic is organized near to every community, which helps the community to access health service and supplement VIT A to their children within the recommended time.

### 4.1. Limitation of the Study

Vitamin A supplementation information was based on the report of the mothers; mothers are usually busy with their routine work and might not remember correctly, which may create a recall bias. Night blindness was only assessed for children age ≥24 months because children <24 months were unable to complain about the problems, which underestimates the true prevalence. All children were examined clinically for an ocular sign of VAD, which is the “tip of the iceberg” that does not tell us subclinical VAD for the children who were normal for an ocular sign of VAD without serum retinol level analysis.

## 5. Conclusion

This study revealed that vitamin A supplementation coverage in the study area was very low as compared with the UNICEF threshold of 70%. On the other hand, the prevalence of Bitot's spot in the study area was higher than that of the cut-off point set by WHO of 1.56%. 6–23 months age of children, high wealth index of the family, good maternal knowledge, the time taken to reach nearby health institution (<30 minutes), taking health education before the campaign in the last six months, and being members of HDA were the main factors associated with VAS coverage in the study area. Increasing the level of maternal knowledge through creating awareness on the health benefits of vitamin A, the consequence of VAD, and food sources is very crucial. Emphasis is laid on the use of the existing health structures at community level like Health Development Army. Being a member of these 1 to 5 networks would significantly enhance their awareness towards a healthier behavior and eventually trigger utilizing the health services consistently.

## Figures and Tables

**Table 1:** Sociodemographic and economic characteristics mothers of children aged 6–59 months in Aleta Chuko woreda, SNNPR, Ethiopia, 2019.

Variables	Frequencies (*n* = 673)	Percent
*Maternal age*		
15–24	237	35.6
25–34	378	56.8
≥35	50	7.5

*Maternal educational status*		
Illiterate	46	6.9
Grades 1–8	421	63.3
Grade 9 and above	198	29.8

*Paternal educational status*		
Illiterate	40	6
Grades 1–8	365	54.9
Grade 9 and above	260	39.1

*Occupation of husband (head of house)*		
Farmer	316	47.5
Small-scale trade	294	44.2
Government employee	46	6.9
Unemployed	5	0.8
Others	4	0.6

*Family size*		
<5	362	54.4
≥5	303	45.6

*Occupation of mother*		
Housewife	525	78.9
Small-scale trade	109	16.4
Government employee	25	3.8
Unemployed	6	0.9

Wealth category		
Rich	221	33.2
Middle	219	33
Poor	225	33.8

*Time taken to reach the nearby health facility*		
<30 minutes	467	70.2
≥30 minutes	198	29.8

**Table 2:** Demographic characteristics of children in 2019.

Variables	Frequencies	Percent
*Sex of children*		
Male	331	49.8
Female	334	50.2

*Age of children*		
6–23	240	36.1
24–59	425	63.9

*Number of under-five children in the house*		
1	582	87.5
>1	83	12.5

*Birth order of children*		
1	243	36.5
2–4	370	55.6
>4	52	7.8

**Table 3:** Vitamin A-related knowledge of mothers with children 6–59 months among mothers or caregivers in Aleta Chuko woreda, 2019.

Variables	Frequencies	Percent
*Correct identification of vitamin A capsule*		
Yes	382	57.4
No	283	42.6

*Know route of VAS*		
Yes	488	73.6
No	175	26.4

*Health benefits of VIT A*		
Prevents disease	194	29.1
Helps in growth	153	23
Prevents night blindness	49	7.4
Others	51	7.7
Does not know	218	32.8

*Consequence of VAD*		
Poor growth	202	30.4
Frequent illness	54	8
Night blindness	33	5
Death	24	3.6
Does not know	352	53

*Listing natural food sources of VIT A*		
Carrots, mango, and papaya	177	26.6
Milk and milk products	141	21.2
Dark-green vegetables	98	14.7
Eggs	50	7.5
Others	5	0.75
Does not know	194	29.2

*Know the recommended schedule of VAS*		
Yes	112	16.8
No	553	83.4

*Know eligible groups for VAS*		
Yes	33	4.9
No	632	95.1

*Malnourished child take VIT A*		
Yes	258	38.8
No	407	60.2

*VAS every six months has a side effect*		
Yes	37	5.6
No	628	94.4

*Overall knowledge*		
Good	150	22.6
Poor	515	77.4

**Table 4:** Health seeking behavior of mothers with children aged 6–59 months in Aleta Chuko woreda, SNNPR, 2019.

Variables	Frequency	Percent
*ANC visit* (*n* *≥* 1)		
Yes	620	93.2
No	45	6.8

*Place of delivery*		
Health facility	529	79.5
Home	136	20.5

*Mother took VITA after delivery*		
Yes	166	25
No	499	75

*Breastfeeding*		
Yes	661	99.4
No	4	0.6

*Member of HDA*		
Yes	167	25.1
No	498	74.9

*Model in HEP*		
Yes	108	16.2
No	557	83.8

*Participated in pregnant mother forum*		
Yes	360	54.1
No	305	45.9

*Children immunization status*		
Immunized all	627	94.3
Partially immunized	38	5.7
Not immunized at all	5	0.8

**Table 5:** Methods of VAS among children aged 6–59 months in Aleta Chuko woreda, SNNPR, 2019.

Vit A supplemented during	Child age in months					
	6–23		24–59		Total	
	Frequency	(%)	Frequency	(%)	Frequency	(%)
Community Heath Day	23	9.5	99	41.1	122	50.6
Immunization session	78	32.4	18	7.5	96	39.8
Home visit	7	2.9	7	2.9	14	5.8
Others			9	3.7	9	3.8

^*∗*^Others are growth monitoring program and sick child visit.

**Table 6:** Vitamin A coverage in the last six months among children aged 6–59 months in Aleta Chuko woreda, 2019.

Characteristics	Vitamin A received in the last six months			
	Yes	Percent	No	Percent
*Child's age in months*				
6–23	108	45	132	55
24–59	133	31.3	292	68.7
Total	241	36.2	424	63.8

*Sex of children*				
Male	121	36.5	210	63.5
Female	120	35.5	214	64.5
Total	241	36.2	424	63.8

**Table 7:** Factors significantly associated with the utilization of VAS among children aged 6–59 months in Aleta Chuko woreda, SNNPR, 2019.

Independent variables	Received vitamin A		COR	AOR (95% CI)
	Yes (%)	No (%)		
*Age of child (months)*				
6–23	108 (45)	132 (55)	1.8 (1.3–2.5)	2.1 (1.4–2.9)^*∗∗∗*^
24–59	133 (31.3)	292 (68.7)	1	1

*Wealth status*				
High	99 (44.8)	122 (55.2)	2.5 (1.7–3.7)	2.3 (1.4–3.8)^*∗∗*^
Middle	87 (39.7)	132 (60.3)	2.1 (1.3–3)	2.7 (1.6–4.5)^*∗∗*^
Poor	55 (24.4)	170 (75.6)	1	1

*Knowledge status*				
Good	90 (60)	60 (40)	3.6 (2.5–5.3)	1.5 (1.2–2.1)^*∗∗*^
Poor	151 (29.3)	364 (70.7)	1	1

*Preference of VAS methods*				
Routine HF VAS	116 (47.9)	126 (52.1)	1.9 (1.1–3.5)	1.5 (0.74–2.8)
Home-to-home VAS	106 (29.1)	258 (70.9)	0.9 (0.5–1.5)	0.9 (0.6–1.7)
CHDs VAS	19 (32.2)	40 (67.8)	1	1

*Member of HDA*				
Yes	103 (61.7)	64 (38.3)	4.2 (2.9–6.1)	2.7 (1.7–4.2)^*∗*^
No	138 (27.7)	360 (72.3)	1	1

*Took health education on VAS in the last six months*				
Yes	69 (66.3)	35 (33.7)	4.5 (2.8–6.9)	3.4 (2.1–5.6)^*∗∗*^
No	172 (30.7)	389 (69.3)	1	1

*Time is taken to reach nearby VAS site*				
<30 minutes	192 (41.1)	275 (58.9)	2.1 (1.5–3.1)	2.5 (1.6–3.8)^*∗∗*^
≥30 minutes	49 (24.7)	149 (75.3)	1	1

*Participated in a pregnant mother's forum*				
Yes	163 (45.3)	197 (54.7)	2.4 (1.7–3.3)	1 (0.6–1.6)
No	78 (25.6)	227 (74.4)	1	1

Note: ^*∗*^indicates significance at *P*< 0.05; ^*∗∗*^indicates significance at *P*< 0.01; and ^*∗∗∗*^indicates significance at *P* < 0.001.

## Data Availability

The datasets generated and/or analyzed during the current study are available from the corresponding author upon reasonable request.

## Abbreviations

AOR: Adjusted odds ratio
CHD: Community Health Day
COR: Crude odds ratio
EDHS: Ethiopia Demographic and Health Survey
EFMoH: Ethiopia Federal Ministry of Health
EPI: Expanded Program on Immunization
HDA: Health Development Army
HEP: Health extension package
IRS: Institutional review board
KAP: Knowledge attitude and practice
PCA: Principal component analysis
UNICEF: United Nations International Children's Emergency Fund
SNNPRS: Southern Nations, Nationalities, and Peoples' Regional State
VAS: Vitamin A supplementation
VAC: Vitamin A capsule
VAD: Vitamin A deficiency
VIT A: Vitamin A
VIF: Variance inflation factor
WHO: World Health Organization.
